# Supplementary material for: Enhanced mitochondrial biogenesis promotes neuroprotection in human pluripotent stem cell derived retinal ganglion cells
Source: Commun Biol. 2023 Feb 24;6:218. doi: 10.1038/s42003-023-04576-w (PMC9957998; doi:10.1038/s42003-023-04576-w)
Supplement: Supplementary file 2 — Supplementary Information [file 42003_2023_4576_MOESM2_ESM.pdf]

Supplementary Figure 1

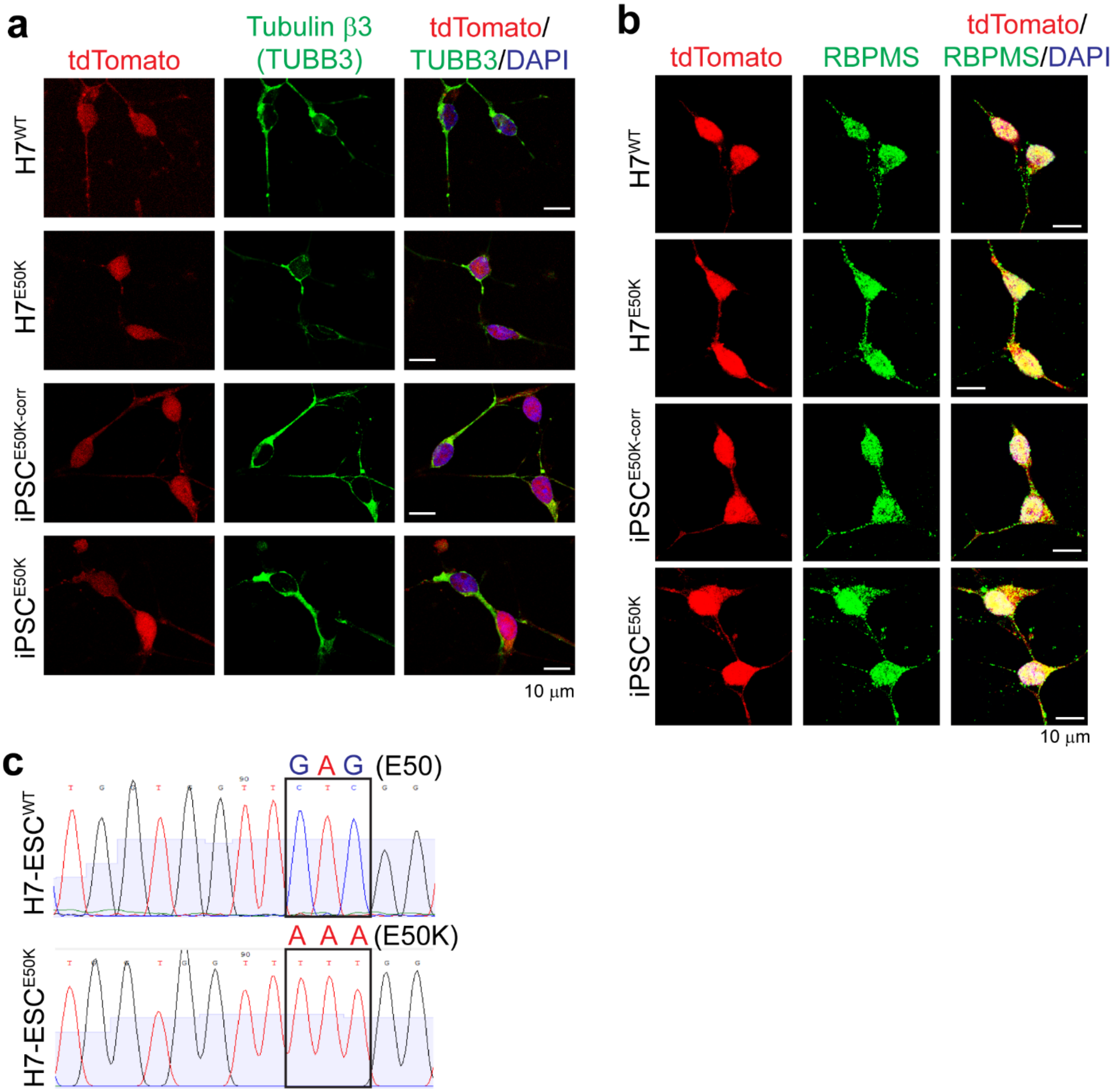

Supplementary Figure 1. hRGC differentiation and CRISPR mutated *OPTN*^{E50K} stem cell lines. (a-b) Shown are confocal immunofluorescence images of hRGCs against **(a)** neuronal marker TUBB3 and **(b)** RGC specific marker RBPMS. Scale bars are 10 µm. **(c)** Sanger sequencing chromatograms show the E50 sequence and the homozygous E50K mutation in the H7-ESCs.

Supplementary Figure 2

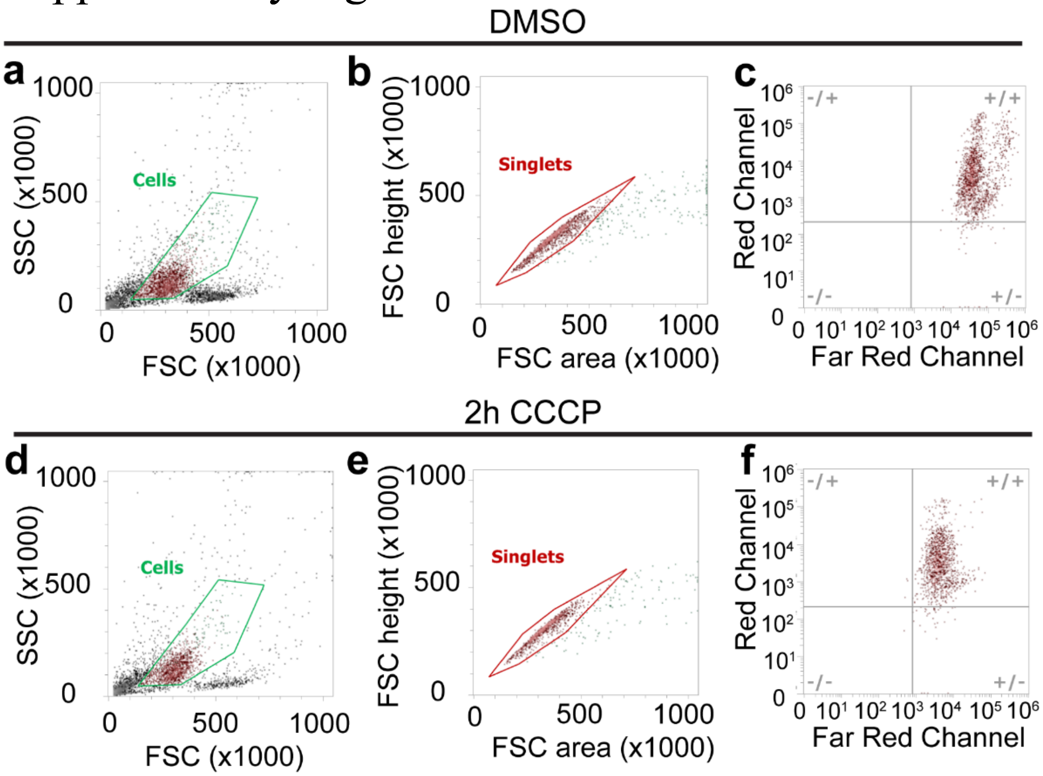

Supplementary Figure 2. Flow cytometry measurements of MTDR labelled mitochondrial mass in hRGCs. Live hRGCs were gated from the total population measured after **(a)** DMSO and **(d)** 2h 10 µM CCCP treatments. From the live cells, **(b, e)** singlet population was then gated, and (**c, f)** subsequently analyzed for average MTDR intensity positive for both tdTomato (red) and MTDR (far red). **(f)** CCCP treatment reduces MTDR positive mitochondrial mass as shown by the left shift of MTDR positive cells.

Supplementary Figure 3

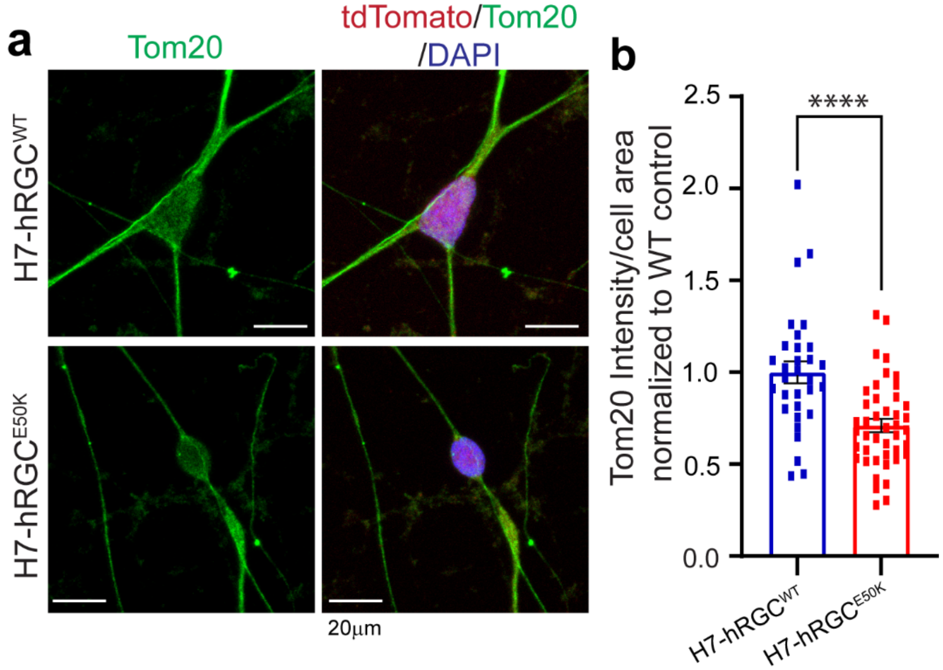

Supplementary Figure 3. Glaucomatous *OPTN^{E50K}* hRGCs possess less mitochondrial mass. **(a)** Representative confocal immunofluorescence images of Tom20, tdTomato, and DAPI of untreated WT and E50K H7-hRGCs. Scale bars are 20 μm. **(b)** Quantification of Tom20 intensity from the sum projections of z-stacks relative to cell area, normalized to WT. Unpaired student's *t-test*. ****, p-value < 0.0001, n=30 cells (3 biological replicates). Error bars are SEM.

Supplementary Figure 4

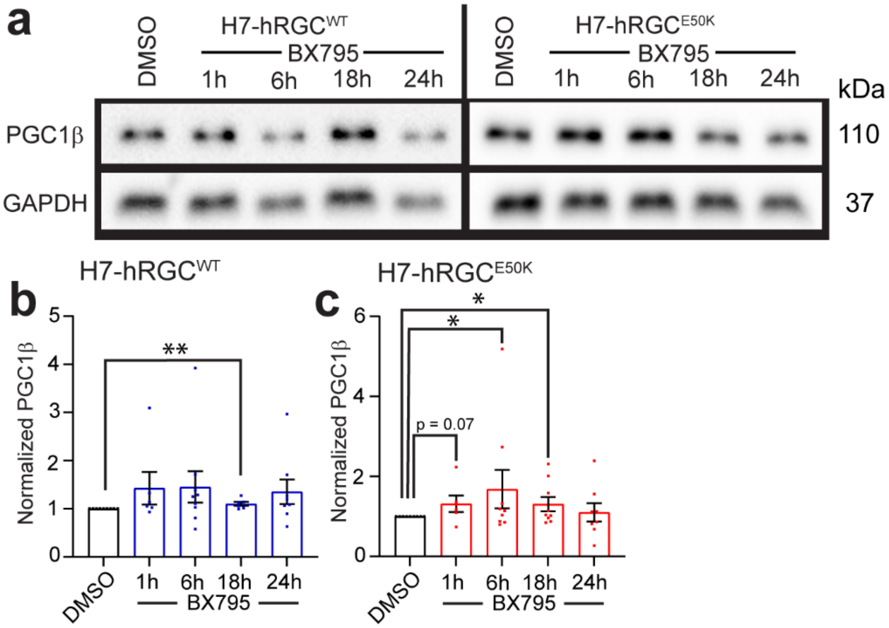

Supplementary Figure 4. TBK1 inhibition by BX795 increases PGC1β level in hRGCs. **(a)** Representative western blot images of H7-hRGCs treated with 1 µg/ml BX795 for the indicated timepoints. **(b, c)** Quantification of average western blot band intensities of PGC1β relative to its GAPDH loading control, then normalized to DMSO control, for **(b)** WT and **(c)** E50K hRGCs. n=6-9. Unpaired student's *t-test* between DMSO and the individual timepoints. * p-value < 0.05, ** p-value < 0.01. Error bars are SEM.

Supplementary Figure 5

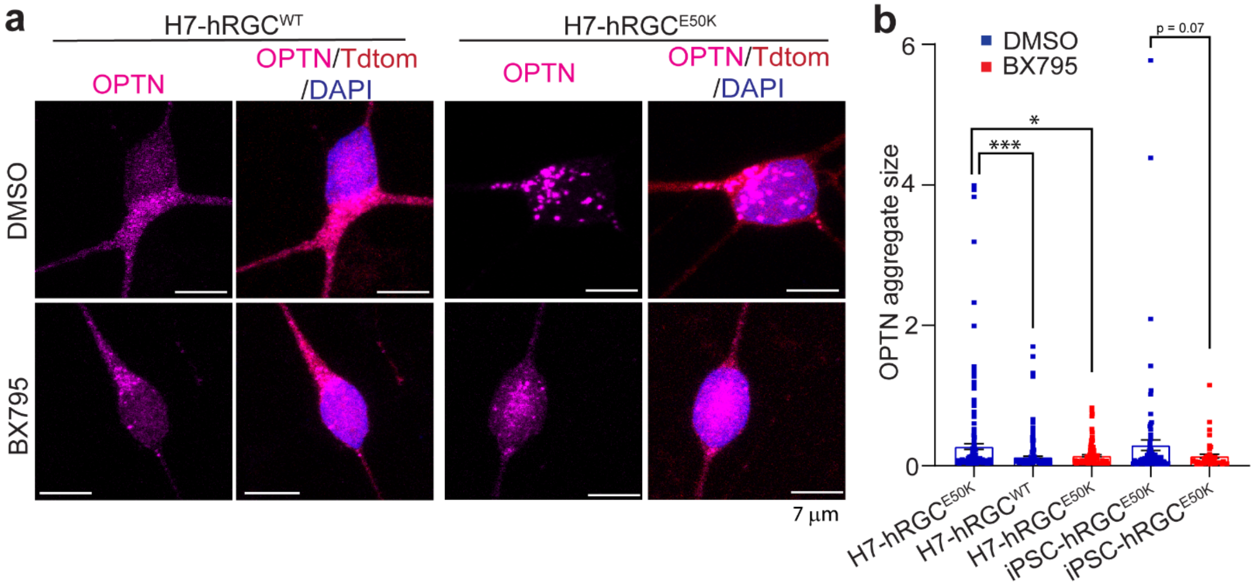

Supplementary Figure 5. BX795 treatment dissolves OPTN^{E50K} aggregates in hRGCs.
(a) Representative confocal immunofluorescence images of OPTN, tdTomato, and DAPI of H7-hRGCs treated with 1 µg/ml BX795 for 24hrs. **(b)** Quantification of OPTN aggregate size from the sum projections of confocal z-stacks. Mann Whitney U test between independent data sets. * p-value <0.05, *** p-value <0.001, n=130-250 aggregates. Error bars are SEM.

Supplementary Figure 6

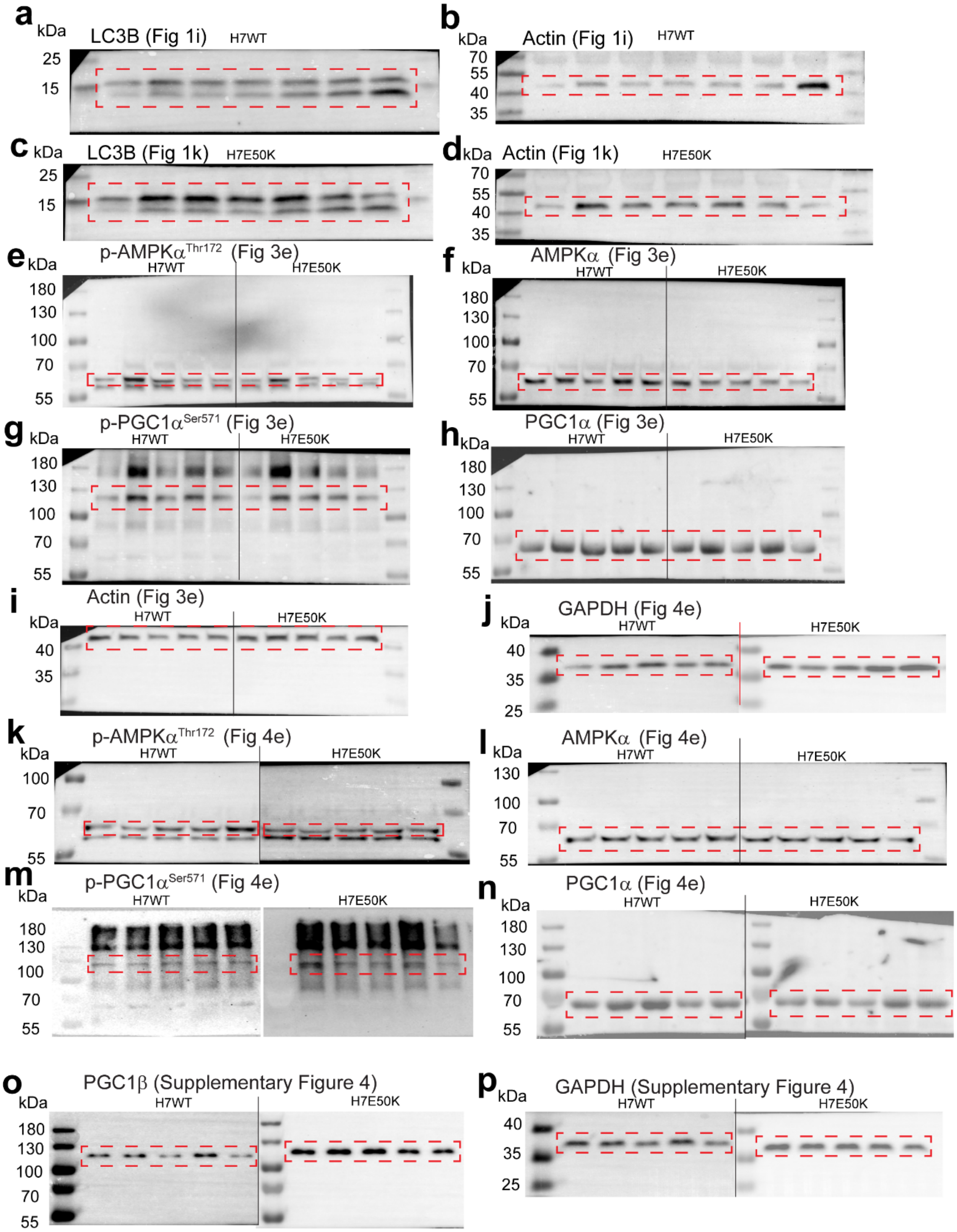

Supplementary Figure 6. Full size images of representative western blots.
Full blot images of western blot membranes from hRGCs shown in previous figures with molecular weight marker. Red boxes represent quantified protein bands.

Supplementary Table 1: list of reagents, software, antibodies, and primers (qPCR)

**REAGENTS AND ASSAY KITS**		
**Reagent**	**Manufacturer**	**Catalog Number**
Matrigel	Corning	CB40230
Gentle Cell Dissociation Reagent	Stem Cell Technology	7174
mTeSR1	Stem Cell Technology	85850
Accutase	Sigma	A6964
Blebbistatin	Sigma	B0560
DMEM/F-12	Thermo Scientific	11330032
Neurobasal media	Thermo Scientific	21103049
GlutaMAX supplement (100X)	Invitrogen	35050061
Antibiotic-Antimycotic	Thermo Scientific	15240062
B27 supplement	Thermo Scientific	17504044
N2 supplement	Thermo Scientific	17502048
Nicotinamide	Sigma	N3376
IDE2	Tocris	4016
Forskolin	Stem cell technologies	3828S
Dorsomorphin	Stem cell technologies	P5499
DAPT	Sigma	D5942
MACS kit	Miltenyi Biotec	130-091-051<br>130-042-108
CD90.2 MicroBeads, mouse	Miltenyi Biotec	130-121-278
Dimethylsulphoxide (DMSO)	Sigma	276855
Carbonyl cyanide-3-chlorophenylhydrazone (CCCP)	Sigma	C2759
BX-795 hydrochloride	Sigma	SML0694
M-PER mammalian protein extraction reagent	Thermo Scientific	78503
Halt protease and phosphatase inhibitor cocktail (100X)	Thermo Scientific	1861281
0.5 M EDTA solution (100X)	Thermo Scientific	1861274
DC Protein Assay Kit II	Bio-Rad	5000112
Laemmli buffer (4X)	Bio-Rad	1610747
PageRuler™ Prestained Protein Ladder	Thermo Scientific	PI26616
BioRad Mini-PROTEAN TGX precast gels	Bio-Rad	4561024<br>4561025
Running buffer (10x Tris/Glycine/SDS)	Bio-Rad	1610732
Transfer buffer (10x Tris/Glycine)	Bio-Rad	1610734
Immuno-blot PVDF membrane	Bio-Rad	1620260
Methanol	Sigma	34860
TBS buffer 20x	VWR	J640
Tween 20	Sigma	P9416
Clarity Max Western ECL substrate	Bio-Rad	1705060
DAPI	Molecular Probes	D1206
MitoTracker™ Deep Red FM (MTDR)	Invitrogen	M22426
Paraformaldehyde 16% solution, EM grade	Electron Microscopy Sciences	15710
Triton-X-100	Sigma	T8787
Donkey Serum	Sigma	D9663
RNeasy Mini Kit	Qiagen	74104

5x all-in-one RT MasterMix (with AccuRTGenomic DNA Removal kit)	Applied biological materials	G492
BlasTaq 2X qPCR MasterMix	Abcam	G891
DNeasy Blood & Tissue Kit	Qiagen	69506
TaqMan Fast Universal PCR Master Mix (2X), no AmpErase UNG	Applied Biosystems	4352042
TaqMan™ Copy Number Reference Assay, human, RNase P	Applied Biosystems	4403326
JC1 dye	Cayman Chemicals	10009172
ApoTox-Glo™ Triplex Assay	Promega	G6320
Seahorse XF Cell Mito Stress Test Kit	Agilent	103015-100
Seahorse XF DMEM medium, pH 7.4	Aglient	103575-100
Seahorse XF 1.0 M glucose solution	Agilent	103577-100
Seahorse XF 100 mM pyruvate solution	Agilent	103578-100
Seahorse XF 200 mM glutamine solution	Agilent	103579-100
Oligomycin from *Streptomyces diastatochromogenes*	Sigma	O4876
Antimycin A from *Streptomyces* sp.	Sigma	A8674
Rotenone	Sigma	R8875
2-Deoxy-D-glucose	Sigma	D8375
Carbonyl cyanide 4-(trifluoromethoxy)phenylhydrazone (FCCP)	Sigma	C2920
**ANTIBODIES**		
**Antibody**	**Manufacturer**	**Catalog Number**
β-actin (rabbit)	Cell signaling technologies	4967S
GAPDH (rabbit monoclonal)	Cell signaling technologies	2118S
PGC1α - N-terminal (rabbit)	Abcam	ab191838
Human Phospho-PGC1α (S571) (rabbit)	R&D Systems	AF6650
AMPKα (rabbit)	Cell signaling technologies	2532S
Phospho-AMPKα (Thr172) (rabbit monoclonal)	Cell signaling technologies	2535T
PGC1β (rabbit monoclonal)	Abcam	ab176328
LC3B (rabbit)	Sigma	L7543
Goat anti-rabbit-IgG1-HRP-linked Ab	Cell signaling technologies	7074S
Optineurin (C-Term) (rabbit polyclonal)	Cayman Chemicals	100000
TOM 20 (mouse)	Santa Cruz	sc-17764
Tubulin β 3 (TUBB3) (mouse)	Biolegend	801202
RBPMS (rabbit)	GeneTex	GTX118619
Donkey anti-mouse Alexa Fluor 488	Invitrogen	A12379
Donkey anti-rabbit Alexa Fluor 647	Invitrogen	A31573
Donkey anti-rabbit Alexa Fluor 488	Invitrogen	A21206
**SOFTWARE AND ALGORITHMS**		
ImageJ	NIH	
Prism version 9	GraphPad	
Zen Microscope Software	Zeiss	
Seahorse Wave Desktop	Agilent	
PrimerBank	https://pga.mgh.harvard.edu/primerbank/	
BioRender	Biorender.com	

**PRIMERS FOR qPCR**		
GAPDH	PrimerBank ID: 83641890b1	F: 5’AAGGTGAAGGTCGGAGTCAAC3’

		R: 5'GGGGTCATTGATGGCAACAATA3'
PGC 1α	PrimerBank ID: 116284374c1	F: 5'TCTGAGTCTGTATGGAGTGACAT3'<br>R: 5'CCAAGTCGTTCACATCTAGTTCA3'
PGC 1β	PrimerBank ID: 289577089c1	F: 5'GATGCCAGCGACTTTGACTC3'<br>R: 5'ACCCACGTCATCTTCAGGGA3'
PRC	PrimerBank ID: 40807451c1	F: 5'CAAGCGCCGTATGGGACTTT3'<br>R: 5'GGAGGCATCCATGTAGCTCT3'
NRF 1	PrimerBank ID: 93141038c1	F: 5'AGGAACACGGAGTGACCCAA3'<br>R: 5'TATGCTCGGTGTAAGTAGCCA3'
NRF 2	PrimerBank ID: 372620347c1	F: 5'TCAGCGACGGAAAGAGTATGA3'<br>R: 5'CCACTGGTTTCTGACTGGATGT3'
ND1 F	https://doi.org/10.1007/s13277-014-2937-2	5'CCTTCGCTGACGCCATAAA3'
ND1 R		5'TGGTAGATGTGGCGGGTTTT3'
ND1 Probe		6FAM-5'TCTTCACCAAAGAGCC3'-MGBNFQ
