# Supplementary material for: Enhanced mitochondrial biogenesis promotes neuroprotection in human pluripotent stem cell derived retinal ganglion cells
Source: Commun Biol. 2023 Feb 24;6:218. doi: 10.1038/s42003-023-04576-w (PMC9957998; doi:10.1038/s42003-023-04576-w)
Supplement: Supplementary file 5 — Reporting Summary [file 42003_2023_4576_MOESM5_ESM.pdf]

nature portfolio

Corresponding author(s): Arupratan Das

Last updated by author(s): 1/30/2023

# Reporting Summary

Nature Portfolio wishes to improve the reproducibility of the work that we publish. This form provides structure for consistency and transparency in reporting. For further information on Nature Portfolio policies, see our Editorial Policies and the Editorial Policy Checklist.

## Statistics

For all statistical analyses, confirm that the following items are present in the figure legend, table legend, main text, or Methods section.

| n/a | Confirmed | |
|---|---|---|
| ☐ | ☒ | The exact sample size (*n*) for each experimental group/condition, given as a discrete number and unit of measurement |
| ☐ | ☒ | A statement on whether measurements were taken from distinct samples or whether the same sample was measured repeatedly |
| ☐ | ☒ | The statistical test(s) used AND whether they are one- or two-sided<br>*Only common tests should be described solely by name; describe more complex techniques in the Methods section.* |
| ☒ | ☐ | A description of all covariates tested |
| ☐ | ☒ | A description of any assumptions or corrections, such as tests of normality and adjustment for multiple comparisons |
| ☐ | ☒ | A full description of the statistical parameters including central tendency (e.g. means) or other basic estimates (e.g. regression coefficient) AND variation (e.g. standard deviation) or associated estimates of uncertainty (e.g. confidence intervals) |
| ☐ | ☒ | For null hypothesis testing, the test statistic (e.g. *F*, *t*, *r*) with confidence intervals, effect sizes, degrees of freedom and *P* value noted<br>*Give P values as exact values whenever suitable.* |
| ☒ | ☐ | For Bayesian analysis, information on the choice of priors and Markov chain Monte Carlo settings |
| ☒ | ☐ | For hierarchical and complex designs, identification of the appropriate level for tests and full reporting of outcomes |
| ☒ | ☐ | Estimates of effect sizes (e.g. Cohen's *d*, Pearson's *r*), indicating how they were calculated |

*Our web collection on statistics for biologists contains articles on many of the points above.*

## Software and code

Policy information about availability of computer code

| | |
|---|---|
| Data collection | ImageJ, Prism Version 9, Zen Microscope Software, Seahorse Wave Desktop, Primer Bank, Attune NxT Software, BioTek Gen5, Image Lab Software (Biorad), Quant Studio (Thermo Fisher) |
| Data analysis | ImageJ, Prism Version 9, Zen Microscope Software, Seahorse Wave Desktop, Microsoft Excel, Attune NxT Software, Quant Studio (Thermo Fisher), BioRender |

For manuscripts utilizing custom algorithms or software that are central to the research but not yet described in published literature, software must be made available to editors and reviewers. We strongly encourage code deposition in a community repository (e.g. GitHub). See the Nature Portfolio guidelines for submitting code & software for further information.

## Data

Policy information about availability of data

All manuscripts must include a data availability statement. This statement should provide the following information, where applicable:

- Accession codes, unique identifiers, or web links for publicly available datasets
- A description of any restrictions on data availability
- For clinical datasets or third party data, please ensure that the statement adheres to our policy

The datasets generated during and/or analyzed during the current study are available from the corresponding author on reasonable request.

# Field-specific reporting

Please select the one below that is the best fit for your research. If you are not sure, read the appropriate sections before making your selection.

☒ Life sciences ☐ Behavioural & social sciences ☐ Ecological, evolutionary & environmental sciences

For a reference copy of the document with all sections, see nature.com/documents/nr-reporting-summary-flat.pdf

# Life sciences study design

All studies must disclose on these points even when the disclosure is negative.

| | |
|---|---|
| Sample size | Sample sizes for this study were based on prior studies that showed statistically significant effects. As such, at least three independent biological repeats were performed for each condition to measure the average mitochondrial mass per cell by flow cytometry, cell viability by fluorescence plate reader and western blot analysis. Gene-expression analysis and mitochondrial DNA copy number measurements by qPCR were performed for each condition on at least three independent biological repeats with three technical repeats for each. Confocal imaging was conducted to analyze mitochondrial level, JC1 intensity, and OPTN aggregates on 10-40 RGC neurons for each condition. Electron Microscopy analysis on mitochondrial morphology was done from 15-24 RGC neurons. Mitochondrial metabolism was performed by Seahorse analyzer for each condition on at least 3 independent biological repeats. |
| Data exclusions | Data were not excluded from analysis, except if experiment failed due to instrumental problem or sample damage. |
| Replication | All experiments were performed with at least three biological repeats and verified reproducibly between different batches of RGC differentiations. |
| Randomization | Confocal imaging and electron microscopy analysis were performed on randomly selected cells equally with no sub-sampling, and thus there was no requirement for randomization. For other analysis such as qPCR, western blot, cell viability, flow cytometry, and seahorse no randomization was used. |
| Blinding | Experiments were performed independently by two authors for validating the conclusions. Quantifications were performed equally for all conditions for a given experiment. |

# Reporting for specific materials, systems and methods

We require information from authors about some types of materials, experimental systems and methods used in many studies. Here, indicate whether each material, system or method listed is relevant to your study. If you are not sure if a list item applies to your research, read the appropriate section before selecting a response.

## Materials & experimental systems

| n/a | Involved in the study | |
|---|---|---|
| ☐ | ☒ | Antibodies |
| ☐ | ☒ | Eukaryotic cell lines |
| ☒ | ☐ | Palaeontology and archaeology |
| ☒ | ☐ | Animals and other organisms |
| ☒ | ☐ | Human research participants |
| ☒ | ☐ | Clinical data |
| ☒ | ☐ | Dual use research of concern |

## Methods

| n/a | Involved in the study | |
|---|---|---|
| ☒ | ☐ | ChIP-seq |
| ☐ | ☒ | Flow cytometry |
| ☒ | ☐ | MRI-based neuroimaging |

## Antibodies

| | |
|---|---|
| Antibodies used | Please see supplemental table S1 for the list of all antibodies used in this study with vendor information and catalog number. |
| Validation | Antibodies used in the manuscript are rigorously validated by multiple publications by other authors in the field. |

## Eukaryotic cell lines

Policy information about cell lines

| | |
|---|---|
| Cell line source(s) | Human embryonic stem cells (H7-hESCs; WiCell, NIH registration #0061).<br>Patient derived de-identified induced pluripotent stem cells (iPSCs) with E50K mutation (iPSC-E50K) (Ohlemacher, S. K. et al, Stem Cells, 2016) and E50K mutation correction (iPSC-E50Kcorr) (VanderWall, K. B. et al, Stem Cell Reports, 2020). |
| Authentication | H7-ESCs and iPSCs used in this work are authenticated by WiCell for their human origin. |


March 2021

| | |
|---|---|
| Mycoplasma contamination | All cell lines are routinely tested for mycoplasma and only used in experiments with mycoplasma negative cells. |
| Commonly misidentified lines (See ICLAC register) | No commonly misidentified cell lines were used. |

# Flow Cytometry

## Plots

Confirm that:

☒ The axis labels state the marker and fluorochrome used (e.g. CD4-FITC).

☒ The axis scales are clearly visible. Include numbers along axes only for bottom left plot of group (a 'group' is an analysis of identical markers).

☒ All plots are contour plots with outliers or pseudocolor plots.

☒ A numerical value for number of cells or percentage (with statistics) is provided.

## Methodology

| | |
|---|---|
| Sample preparation | hRGCs were differentiated and purified from human pluripotent stem cells as described in the methods. Purified hRGCs were labeled with mitochondria specific MTDR dye. After treatments, hRGC were dissociated into single cell suspensions using accutase for flow cytometry measurements. |
| Instrument | Attune NxT (ThermoFisher) |
| Software | Attune NxT Software |
| Cell population abundance | Analysis were done using 10,000 cells per condition. No cell sorting has been performed. |
| Gating strategy | Gating strategy has been explained in detail in supplemental figure 2. |

☒ Tick this box to confirm that a figure exemplifying the gating strategy is provided in the Supplementary Information.
